# Transformational leadership of physical education instructors and university students' satisfaction with online classes

**DOI:** 10.3389/fpsyg.2023.1259218

**Published:** 2023-10-12

**Authors:** Angelita Bautista Cruz, Hyun-Duck Kim

**Affiliations:** ^1^Department of Physical Education, Keimyung University, Daegu, South Korea; ^2^Department of Sport Marketing, Keimyung University, Daegu, South Korea

**Keywords:** online physical education, university students, transformational leadership theory, physical education teacher, Philippines, student learning outcome

## Abstract

This study examined the relationship between the transformational leadership of PE instructors and students' satisfaction in an online PE class. In particular, it aimed to investigate whether the PE instructors' transformational leadership behaviors could predict students' satisfaction toward the class, their PE teacher, and their health and fitness. Furthermore, this study explored these relationships in male and female students. A total of 448 university students (male = 228; female = 220) between the ages of 18 and 22 participated in the study. The results revealed a positive relationship between the transformational leadership behaviors of PE instructors and students' satisfaction with online PE classes. Moreover, male and female students' satisfaction with the teaching of their PE instructor, feelings of fun and enjoyment, and perception of improved health and fitness in their online PE classes varied greatly as they perceived specific behaviors of transformational leadership from their PE instructors. These findings demonstrate that PE instructors play an important role as (online) classroom leaders in enhancing students' satisfaction with online learning. Therefore, PE instructors should be mindful to demonstrate transformational leadership to improve their effectiveness when conducting online classes.

## 1. Introduction

Teachers are regarded as classroom leaders. They plan and conduct appropriate lessons, effectively communicate the curriculum content, and provide appropriate support and encouragement for students to achieve their learning goals (Nadelson et al., 2020). In physical education (PE), aside from these general responsibilities, PE instructors teach and demonstrate fundamental movement and sports-related skills, games, and fitness exercises that are generally conducted in schools' indoor or outdoor facilities (Shirotriya and Beighle, 2022). They also motivate students to be active not only during the PE period but also outside PE hours. Indeed, PE teachers have distinctive functions and responsibilities that exemplify their leadership position when teaching. However, because of the unprecedented global impact of the COVID-19 pandemic, face-to-face PE classes have shifted to online instruction (Cruz et al., 2022). This change in online educational instruction has made teaching PE lessons more challenging (Chan et al., 2021; Korcz et al., 2021; Yu and Jee, 2021), particularly when teaching games, exercises, and sports that require specific equipment and/or facilities and interactions among classmates (Chan et al., 2021; Kim et al., 2021). While the majority of schools have reverted to face-to-face PE classes, a number of academic institutions still conduct PE classes in an online format. Therefore, this topic requires continuous examination. In particular, PE teachers' leadership approaches in teaching online PE classes and how these leadership behaviors affect class-related outcomes in students merit further research. Understanding the impact of transformational leadership and student outcomes in an online setting could help PE teachers improve their teaching and leadership approaches. Such improvement will enable them to effectively guide and motivate their students in learning PE concepts and movement skills even in an online PE classroom environment.

The role of PE teachers as leaders who could facilitate students' class-related outcomes can be viewed using the transformational teaching approach. Transformational teaching is a contemporary approach to classroom learning instruction (Slavich and Zimbardo, 2012). It promotes students' learning attitudes, values, skills, and beliefs and develops their personal growth by establishing dynamic interrelationships among teachers, students, and a shared body of knowledge. Grounded in transformational leadership (Bass, 1998), transformational teaching emphasizes the role of instructors as motivational leaders who influence students by displaying positive traits and behaviors inside and outside the classroom (idealized influence) and conveying clear and realistic goals that students acknowledge as meaningful (inspirational motivation). Transformational teaching also underscores teachers who stimulate students' intellectual capacity by challenging them to find solutions to problems from various perspectives (intellectual stimulation), listening to students' needs and concerns, and providing support when needed (individualized consideration). Therefore, when PE teachers demonstrate the important elements of transformational behaviors, such as acting as role models for students, sharing the goals and objectives of each PE lesson with students, stimulating students' cognitive skills through games and sports, and providing social support and positive reinforcement, students' personal growth and learning outcomes (i.e., beliefs and attitudes) could potentially be enhanced.

Several studies have examined the relationship between teachers' transformational behaviors and class-related outcomes. For instance, Noland and Richards (2014) found that transformational teaching significantly predicts student learning outcomes. In particular, all four elements of transformational teaching show significant relationships with students' affective learning. They concluded that transformational leadership practices in the classroom positively influence students' positive attitudes toward the subject. Harvey et al. (2003) found that students' trust, respect, and satisfaction toward their instructors are positively associated with the instructors' transformational behaviors. Harrison (2011) examined university instructors' leadership behaviors and their impacts on student outcomes and found that instructors' transformational leadership behaviors are significant predictors of students' perceptions of instructor credibility, cognitive learning, and affective learning. Moreover, in a study conducted in PE settings, Beauchamp et al. (2014) found that transformational teaching has a direct effect on students' affective attitudes, suggesting that students' enjoyment of PE can be enhanced when their PE teachers demonstrate transformational behaviors. Wei and Jianhao (2023) revealed that the transformational leadership of PE teachers is positively associated with students' exercise adherence and physical efficacy. Cho et al. (2013) examined the influence of PE teachers' transformational leadership on teacher trust and class satisfaction and found that charisma and intellectual stimulation are positive predictors of teacher trust. They also revealed that students' satisfaction with class evaluations and sense of accomplishment are positively predicted by teachers' transformational leadership.

Overall, the results of these previous studies highlight the influence of teachers' transformational leadership on students' learning outcomes. However, knowledge is still scarce regarding the relationship between transformational leadership and students' learning outcomes in an online education setting, especially in online PE environment. Given the changes in the educational landscape to online education in general and PE in particular because of the COVID-19 pandemic, the role of PE teachers in facilitating student learning has become increasingly pertinent. Therefore, efforts must be exerted to expand the knowledge on how PE teachers can be more effective in enhancing students' learning outcomes in an online PE environment, especially when face-to-face classes are not viable.

Accordingly, the current study aims to examine the relationship between PE teachers' transformational leadership and students' satisfaction in an online PE class. In particular, we investigate the elements of transformational leadership that could predict students' satisfaction toward the class, their PE teacher, and their health and fitness. We also analyze these relationships in male and female students.

## 2. Materials and methods

### 2.1. Participants

A total of 448 university students participated in this study. Specifically, the participants comprised 228 (50.9%) male students and 220 (49.1%) female students, and their ages ranged from 18 to 22 years. They were recruited from a private university located in the second-largest metropolitan area in the Philippines during the second semester of 2022–2023. The students were enrolled in different PE courses, such as traditional Filipino games, modern dance, line dance, movement enhancement, and fitness exercises.

### 2.2. Assessment tools

#### 2.2.1. Transformation leadership

The transformational leadership behaviors of PE instructors, as perceived by students, were assessed using the Transformational Teaching Questionnaire (TTQ) (Beauchamp et al., 2010). The TTQ comprises 16 items divided into 4 subscales: idealized influence (e.g., My PE instructor treats me in ways that build my respect), inspirational motivation (e.g., My PE instructor demonstrates that s/he believes in me), intellectual stimulation (e.g., My PE instructor creates lessons that encourage me to think), and individualized consideration (e.g., My PE instructor tries to help students who might be struggling). The survey used a 5-point Likert scale with response ratings of 0, “not at all”; 1, “once in a while”; 2, “sometimes”; 3, “fairly often”; and 4, “frequently.” Previous studies have supported the measurement tool's psychometric properties (Beauchamp et al., 2010; Rezaei Sharif and Ebrahimkhani, 2018; Trigueros et al., 2020; Wei and Jianhao, 2023). The internal reliability scores for this study were 0.805, 0.893, 0.824, and 0.826 for individualized influence, inspirational motivation, intellectual stimulation, and individualized consideration, respectively.

#### 2.2.2. PE class satisfaction

The students' satisfaction with their PE class, PE teacher, and perceived health and fitness improvement were measured using selected items from the Physical Activity Class Satisfaction Questionnaire (PASCSQ) (Cunningham, 2007), which was adapted for use in online PE classes for this study. The students were asked to rate their level of satisfaction with the aspects of their online PE class, namely, fun and enjoyment (e.g., My overall enjoyment in the online class), teaching (e.g., The clarity of the PE instructor's lessons), and improvement of health and fitness (e.g., The physical workout I receive in this online PE class). The survey had 14 items and used an 8-point Likert scale ranging from 1 (not satisfied) to 8 (very satisfied). The psychometric properties of this tool have been supported in previous studies (Cunningham, 2007; Filippou et al., 2021; La Rotta et al., 2021). The internal reliability scores for this study were 0.929, 0.947, and 0.954 for teaching, fun and enjoyment, and improvement of health and fitness, respectively.

### 2.3. Procedure

This study used a quantitative cross-sectional design, and the respondents were recruited using convenience sampling. The primary author contacted PE instructors, explained the study's purpose, and requested permission to involve their students in the survey. Upon obtaining approval, students enrolled in various PE courses received a QR code and were asked to complete an online survey using Google Forms. The survey included an introduction to the study, information about their rights as participants, assurances of anonymity and confidentiality, and informed consent to participate in the study. Only students who provided informed consent (by clicking on the “Agree” option) were able to proceed to the series of questionnaires on transformational leadership, PE satisfaction, and demographic information. Data were collected near the end of the second semester (April 2023) to ensure that students had acquired sufficient knowledge and experience to assess their PE instructors' behavior during online sessions and their online PE class. The survey took ~15–20 mins to complete. This study complied with the American Psychological Association's ethical standards for human treatment and the ethical guidelines of the appropriate ethics committee in the Philippines.

### 2.4. Data analysis

The students' responses were recorded in Excel. Data screening was conducted to remove missing, duplicate, incorrect, and incomplete data. Out of the 498 collected survey forms, 448 were deemed valid. Descriptive statistics and multiple regression analyses were performed to respectively examine the nature of the data and to determine which transformational leadership behaviors would best predict PE satisfaction. Next, the data were stratified by sex, and a multiple linear regression was performed again to identify which transformational leadership behaviors would best predict PE satisfaction in male and female students. IBM SPSS statistical software version 28 was used to perform the relevant analyses, and the statistical significance was set at *p* < 0.05.

## 3. Results

Table 1 shows the means and standard deviations of PE teachers' transformational leadership and students' PE satisfaction. The mean scores for transformational leadership behavior were consistently above 3.00, suggesting that the university students perceived their PE teachers to display transformational leadership behaviors fairly often when conducting online PE classes. Meanwhile, the mean scores for the dimensions of PE satisfaction were above 6.00, suggesting that the students were moderately satisfied with the teaching of their PE instructors, the pleasure and enjoyment they experienced in the online PE class, and the belief that the online PE class helped improve their health and fitness.

**Table 1:** Descriptive statistics of relevant variables.

**Variable**	**Mean**	**SD**
**Transformational leadership**		
Idealized influence	3.30	0.69
Inspirational motivation	3.41	0.70
Intellectual stimulation	3.12	0.79
Individualized consideration	3.30	0.72
**Satisfaction**		
Teaching	6.90	1.11
Fun and enjoyment	6.67	1.35
Health and fitness	6.52	1.30

Table 2 shows the regression analyses of transformational leadership behaviors and different dimensions of PE satisfaction. Teaching satisfaction was regressed on the four elements of transformational leadership. The overall regression was significant: *F*_(4, 443)_ = 43.365, *p* < 0.001. The model explained ~28% of the variance in teaching satisfaction (adjusted R^2^ = 0.275). Table 2 provides information on the regression coefficients of the predictors. Idealized influence and individualized consideration were significant predictors with a positive relationship with teaching satisfaction.

**Table 2:** Multiple regression analysis of transformational leadership predicting PE satisfaction.

**Variable**	**95% CI**					
	**B**	**LB**	**UB**	β	***t***	***p***
**Satisfaction—teaching**						
Idealized influence	0.445	0.171	0.718	0.274	3.195	0.001^**^
Inspirational motivation	0.247	−0.016	0.509	0.155	1.847	0.065
Intellectual stimulation	−0.064	−0.283	0.155	−0.045	−0.573	0.567
Individualized consideration	0.270	0.027	0.514	0.175	2.181	0.030^*^
**Satisfaction–fun and enjoyment**						
Idealized influence	0.284	−0.067	0.634	0.144	1.591	0.112
Inspirational motivation	−0.032	−0.368	0.304	−0.016	−0.185	0.853
Intellectual stimulation	0.270	−0.011	0.551	0.157	1.891	0.059
Individualized consideration	0.372	0.060	0.684	0.198	2.345	0.019^*^
**Satisfaction–health and fitness**						
Idealized influence	0.319	−0.021	0.658	0.168	1.847	0.065
Inspirational motivation	−0.117	−0.443	0.208	−0.063	−0.708	0.479
Intellectual stimulation	0.349	0.077	0.622	0.211	2.524	0.012^*^
Individualized consideration	0.267	−0.035	0.569	0.148	1.735	0.083

CI, confidence interval; LB, lower bound; UB, upper bound; ^*^p < 0.05; ^**^p < 0.01.

Fun and enjoyment was regressed on the four elements of transformational leadership. The overall regression was significant: *F*_(4, 443)_ = 28.521, *p* < 0.001. The model explained ~21% of the variance in teaching satisfaction (adjusted R^2^ = 0.198). Table 2 provides information on the regression coefficients of the predictors. Individualized consideration was the only significant predictor with a positive relationship with fun and enjoyment.

Health and fitness improvement satisfaction was regressed on the four elements of transformational leadership. The overall regression was significant: *F*_(4, 443)_ = 26.506, *p* < 0.001. The model explained ~19% of the variance in teaching satisfaction (adjusted R^2^ = 0.186). Table 2 provides information on the regression coefficients of the predictors. Intellectual stimulation was the only significant predictor with a positive relationship with health and fitness improvement.

Table 3 shows the regression analyses of transformational leadership behaviors and the different dimensions of PE satisfaction among male and female students. The teaching satisfaction of male and female students was regressed on the four elements of transformational leadership. The regression results were significant for both sexes (males: *F*_(4, 223)_ = 28.244, *p* < 0.001; females: *F*_(4, 215)_ = 16.887, *p* < 0.001). The models explained ~34 and 24% of the variances in teaching satisfaction among male and female students, respectively. Based on the regression coefficients, idealized influence and individualized consideration were significant predictors with a positive relationship with teaching satisfaction in males. As for females, only idealized influence was a significant positive predictor of teaching satisfaction.

**Table 3:** Multiple regression analysis for transformational leadership predicting PE satisfaction in male and female students.

	**95% CI**					
	**B**	**LB**	**UB**	β	***t***	***p***
**Satisfaction–teaching**						
Male						
Idealized influence	0.494	0.102	0.886	0.286	2.485	0.014^*^
Inspirational motivation	0.298	−0.072	0.669	0.182	1.587	0.114
Intellectual stimulation	−0.284	−0.607	0.039	−0.188	−1.730	0.085
Individualized consideration	0.534	0.200	0.868	0.318	3.149	0.002^**^
Female						
Idealized influence	0.438	0.054	0.821	0.292	2.250	0.025^*^
Inspirational motivation	0.171	−0.209	0.551	0.111	0.886	0.377
Intellectual stimulation	0.130	−0.166	0.426	0.100	0.868	0.386
Individualized consideration	0.021	−0.343	0.384	0.015	0.114	0.910
**Satisfaction–fun and enjoyment**						
Male						
Idealized influence	0.240	−0.278	0.757	0.113	0.912	0.363
Inspirational motivation	0.234	−0.255	0.723	0.116	0.944	0.346
Intellectual stimulation	−0.027	−0.454	0.399	−0.015	−0.127	0.899
Individualized consideration	0.614	0.173	1.055	0.298	2.746	0.007^*^
Female						
Idealized influence	0.347	−0.124	0.818	0.193	1.452	0.148
Inspirational motivation	−0.372	−0.839	0.095	−0.201	−1.570	0.118
Intellectual stimulation	0.534	0.170	0.898	0.341	2.895	0.004^**^
Individualized consideration	0.195	−0.252	0.641	0.115	0.858	0.392
**Satisfaction–health and fitness**						
Male						
Idealized influence	0.329	−0.189	0.848	0.158	1.251	0.212
Inspirational motivation	0.163	−0.327	0.653	0.083	0.656	0.513
Intellectual stimulation	−0.042	−0.470	0.386	−0.023	−0.194	0.846
Individualized consideration	0.506	0.064	0.948	0.251	2.257	0.025^*^
Female						
Idealized influence	0.321	−0.108	0.749	0.191	1.474	0.142
Inspirational motivation	−0.475	−0.901	−0.050	−0.275	−2.204	0.029^*^
Intellectual stimulation	0.704	0.373	1.035	0.482	4.194	0.001^**^
Individualized consideration	0.098	−0.309	0.504	0.062	0.475	0.635

CI, confidence interval; LB, lower bound; UB, upper bound; ^*^p < 0.05; ^**^p < 0.01.

Male and female students' fun and enjoyment was regressed on the four elements of transformational leadership. The regression results were significant for both sexes (males: *F*_(4, 223)_ = 16.949, *p* < 0.001; females: *F*_(4, 215)_ = 13.740, *p* < 0.001). The models explained ~23 and 20% of the variances in teaching satisfaction among male and female students, respectively. Based on the regression coefficients, only individualized consideration was a significant predictor with a positive relationship with fun and enjoyment in males. As for females, only intellectual stimulation was a significant positive predictor of fun and enjoyment.

The male and female students' health and fitness satisfaction was regressed on the four elements of transformational leadership. The regression results were significant for both sexes (males: *F*_(4, 223)_ = 13.512, *p* < 0.001; females: *F*_(4, 215)_ = 17.106, *p* < 0.001). The models explained ~20 and 24% of the variances in health and fitness satisfaction in male and female students, respectively. Based on the regression coefficients, only individualized consideration was a significant predictor with a positive relationship with health and fitness satisfaction in males. As for females, inspirational motivation and intellectual stimulation were significant and positive predictors of health and fitness satisfaction.

## 4. Discussion

This study examined the relationship between the transformational leadership of PE teachers and students' satisfaction with online PE classes. In particular, we investigated the elements of transformational leadership that could predict students' satisfaction toward the class, their PE teachers, and their overall health and fitness. We also examined these relationships in male and female students. Overall, the results showed that the university students who attended online PE courses perceived their PE instructors as demonstrating transformational behaviors fairly often. They also reported that they were relatively satisfied with the conduct of their online PE classes.

### 4.1. PE instructors' transformational leadership and students' satisfaction in online PE classes

The regression analysis revealed a significant positive relationship between transformational leadership and PE class satisfaction. In other words, during online PE classes, students who perceive that their PE instructors care for them, demonstrate movement skills well, encourage every student to do their best to achieve class objectives, and let students think of different strategies to solve tactical-related activities are more likely to feel satisfied with the teaching of their PE instructors, consider the online PE class fun and enjoyable, and perceive that the class can help improve their health and fitness. This finding supports those of previous studies that found a positive relationship between the transformational leadership of PE teachers and student outcomes (Beauchamp et al., 2014; Jiang and Jia, 2018; Castillo et al., 2020; Wei and Jianhao, 2023). It also extends the knowledge not only in the education literature but also in the PE teaching literature by examining PE teachers' transformational leadership teaching and how it affects class-related outcomes in students in an online environment. Moreover, the results showed that the distinct elements of transformational leadership significantly predicted PE satisfaction. In particular, idealized influence and individualized consideration were significant positive predictors of teaching satisfaction while individualized consideration and intellectual stimulation were significant positive predictors of fun and enjoyment and health and fitness satisfaction, respectively. This finding suggests that when students perceive that their PE instructor attends to their individual needs and considers their strengths and weaknesses, they are more likely to enjoy and have fun in their online PE classes. In addition to prior leadership behavior, when students observe that their PE teacher behaves in an ethical way and guides them through actions, they tend to feel more satisfied with their instructor's level of teaching. Finally, when students perceive that their PE instructor creates new learning opportunities and develops their critical thinking skills through PE lessons, they are more likely to perceive that their health and fitness is enhanced because of their participation in the online PE class. This result partially supports the previous findings (Cho et al., 2013) that the distinct transformational leadership behaviors of PE teachers significantly affect the PE satisfaction of students. The difference in the results may be due to the measurement tools used, the study participants, and the PE setting (online PE vs. traditional PE classes). As the present study only assessed three dimensions of satisfaction of the PASCSQ, other dimensions of this satisfaction scale should be examined to gain a better understanding of how the transformational leadership of PE teachers affects students' satisfaction in online settings. Nevertheless, the findings are in accordance with the concept of transformational teaching, in which positive learning outcomes would be expected from students when teachers demonstrate transformational behavior.

### 4.2. PE instructors' transformational leadership and male and female students' satisfaction in online PE classes

In addition to examining the association between PE instructors' transformational leadership and students' satisfaction in general, we investigated how the transformational leadership behaviors of PE instructors affect male and female students' levels of satisfaction with their online PE classes. Based on the regression analyses, male students' satisfaction with the fun and enjoyment of their PE classes and their perceived health and fitness were significantly affected only by the individualized consideration element of transformational leadership. These findings indicate that when PE instructors support and encourage students and foster open communication, male students would tend to feel more joyful and experience more fun in online PE classes and think that their health and fitness have improved as a result of perceiving this transformational leadership behavior. Meanwhile, individualized consideration and idealized influence significantly affected the male students' teaching satisfaction. That is, male students would tend to report higher levels of satisfaction with the PE instructor's teaching behaviors when they perceive that their PE instructor acts as a role model, treats students in ways that build respect and trust, and cares about knowing every student in the class.

According to the regression analyses of female students, certain elements of transformational leadership had significant effects on female students' PE satisfaction. Idealized influence was the sole significant predictor of teaching satisfaction while intellectual stimulation was the only predictor of fun and enjoyment. Meanwhile, inspirational motivation and intellectual stimulation were significant predictors of satisfaction with health and fitness. These findings suggest that female students' satisfaction levels are likely to increase when they observe that their PE instructors frequently demonstrate these transformational behaviors. That is, female students would tend to be more satisfied with the PE instructor's teaching when they perceive that the instructor creates a PE class with a shared vision and personifies a skilled and knowledgeable PE educator. In addition, female students are more likely to think that their PE classes are fun and enjoyable when they perceive that their PE instructors provide challenging tasks and encourage multiple perspectives to solve problems. Finally, female students are likely to experience improvement in their health and fitness as a result of attending their online PE classes when they perceive that their PE instructors motivate them to think out of the box, promote creativity and curiosity in the class, and inspire them to achieve PE lessons' goals and objectives.

Overall, the findings demonstrate that similarities and differences in male and female students' appraisals of their learning outcomes (e.g., satisfaction) in online PE classes could be affected by the PE instructors' display of transformational leadership behaviors and how the students perceive these behaviors. This notion is in accordance with the multidimensional model of sports leadership (Chelladurai, 2007), which states that desired outcomes (e.g., satisfaction) are expected when the perceived behaviors of the leader that are deemed appropriate for a certain situation are congruent with the preferences of the followers. Moreover, these actual, required, and preferred leadership behaviors are influenced by various characteristics of the situation, coach, and members. In line with this model, the male and female students' degree of satisfaction with certain dimensions of their PE experiences appeared to be the result of their perceptions of certain leadership behaviors that matched their personal preferences for these specific behaviors. In studies conducted in sports, it was found that males reported stronger preferences for training and instruction, autocratic behavior and social support (Horn, 2002; Witte, 2011) while females expressed higher preferences for democratic decision-making style, positive feedback and situational consideration (Horn, 2002; Witte, 2011). Following this idea in the present study, when PE instructors frequently displayed the appropriate teaching behaviors when conducting online PE class, and these behaviors were congruent with the preferences of male and female students, students tended to feel satisfied with their online PE experiences. Therefore, this result highlights that learning outcomes, that is, satisfaction with online PE, can result from the interaction among the characteristics of the PE instructor, student, and class context. Moreover, this finding emphasizes students' sex as a potential variable in the transformational leadership–satisfaction relationship in the PE context. A previous study in the sports context supports this claim (Kim and Cruz, 2022).

This study also underscores the importance of PE teachers' demonstration of transformational leadership behaviors in enhancing student satisfaction in online PE classes. This is achieved by altering students' beliefs and attitudes toward the classes and fostering positive and dynamic relationships among students, and in turn contributing to their personal growth. Considering that studies conducted in traditional PE environment have shown that transformational leadership of teachers is positively related to other learning outcomes in students, such as motivation (Jiang and Jia, 2018; Castillo et al., 2020), expectancy value in PE (Kim et al., 2017), exercise adherence (Wei and Jianhao, 2023), and physical activity participation (Beauchamp and Morton, 2011; Castillo et al., 2020), it is plausible that these learning outcomes could also be facilitated in an online environment and therefore worthy to be explored in future investigations.

### 4.3. Theoretical contributions and implications

To the best of our knowledge, this study is the first to examine the relationship between PE teachers' transformational leadership and student outcomes (PE satisfaction) in an online setting as well as to investigate this relationship between male and female students. Hence, the findings extend the body of knowledge in the domains of physical education, leadership, and online teaching by providing empirical evidence of the effectiveness of transformational leadership and its elements in enhancing students' satisfaction with their online PE experiences. The findings can also contribute to the academic community, especially for the professional development of physical education instructors who have difficulties in creating an effective and engaging online PE environment for students. Learning the principles of transformational leadership by attending leadership seminars or training workshops can assist PE instructors on how to incorporate appropriate leadership strategies into their teaching approaches, and in turn, lead to better student motivation and engagement in an online PE classroom setting. From a practical perspective, PE instructors should employ effective strategies when teaching and managing their students' learning by frequently demonstrating transformational leadership and teaching behaviors to sustain university students' attention and motivation and to further enhance their satisfaction with their online PE courses. For instance, given individualized consideration being a significant predictor of students' fun and enjoyment, PE instructors should be mindful of students' individual concerns and challenges when attending online PE classes and try to assist them whenever possible. In the Philippines, for example, technological literacy and competency, learning environment, and physical discomfort have been identified as challenges that university students experience during online learning (Barrot et al., 2021). Therefore, PE instructors should be more considerate when students cannot attend synchronous sessions due to internet connection issues. They should also consider extending the submission dates of projects/activity logs for students who may struggle to complete tasks because of a lack of space or equipment to perform such tasks at home. Meanwhile, for PE instructors aiming to create a pleasurable online PE experience for female students, they should focus on innovative and interesting online activities that would stimulate female students' curiosity and learning because the intellectual stimulation element of transformational behavior was found to greatly affect female students' fun and enjoyment. Some activities in the movement enhancement PE classes may include finding things in the house that can be used as alternative equipment for developing muscular endurance or utilizing movement analysis software to provide constructive feedback on movement performance.

### 4.4. Limitations

This study has several limitations. First, student satisfaction is considered one of the main outcomes of education and can be measured in various ways. As the present study assessed only three dimensions of satisfaction (PE instructor's teaching, fun and enjoyment, and health and fitness), we suggest that other dimensions of satisfaction, such as social interaction and cognitive development, be examined to gain a better understanding of how the transformational leadership of PE teachers affects the other dimensions of satisfaction among students attending online PE classes. Second, we recognize the presence of potential response and sampling biases in our data collection process, which may be attributed to the chosen survey. To mitigate potential response bias, we took measures to ensure the clarity and comprehensibility of the questionnaire instructions, maintained participant anonymity, and adapted item statements from validated measurement tools to elicit accurate and honest responses from the participants. Additionally, it is important to note that our sample consisted solely of university students, which limits the generalizability of our study's findings to a broader population. Therefore, we recommend expanding the scope of future studies by including participants from different age groups or educational levels. Third, other personal and situational factors that may mediate or moderate the relationship between PE instructors' transformational leadership and student satisfaction were not examined in the present study. Hence, future studies should consider these variables such as PE instructors' teaching experience, technological ability, and gender as well as students' fitness level, self-efficacy, and academic workload. Finally, because the study adopted a cross-sectional approach, causal relationships could not be claimed; therefore, longitudinal studies should be conducted in the future to supplement the findings of the present investigation.

### 4.5. Conclusion

The current study found that when university students perceived their PE instructors to demonstrate transformational leadership behaviors when conducting online PE classes, they felt relatively satisfied with their online PE experience. Furthermore, the transformational leadership of PE instructors, particularly their individualized consideration, idealized influence, and intellectual stimulation, promoted the university students' PE satisfaction in the online learning setting. Finally, the male and female students' satisfaction with the teaching of their PE instructor, feelings of fun and enjoyment, and perception of improved health and fitness in their online PE classes varied greatly as they perceived specific behaviors of transformational leadership from their PE instructors. Overall, the findings confirm the positive effects of transformational leadership behaviors on students' affective learning outcomes. Thus, PE instructors are encouraged to demonstrate transformational leadership to improve their effectiveness when conducting online PE classes.

## Data availability statement

The raw data supporting the conclusions of this article will be made available by the authors, without undue reservation.

## Ethics statement

Ethical approval was not required for the studies involving humans because relevant officials and PE instructors reviewed and approved the study protocol. The studies were conducted in accordance with the local legislation and institutional requirements. The participants provided their written informed consent to participate in this study.

## Author contributions

AC: Conceptualization, Formal analysis, Writing—original draft, Writing—review and editing. H-DK: Conceptualization, Writing—original draft, Writing—review and editing.
